# Tetany in a Young Female Not Resulting From Hypocalcemia

**DOI:** 10.7759/cureus.43521

**Published:** 2023-08-15

**Authors:** Majed Alanazi, Abdulrhman Alabdulgader, Abdulaziz Alotaibi, Ibrahim Bin Ahmed, Mansour Maskati

**Affiliations:** 1 Internal Medicine, King Abdulaziz Medical City Riyadh, Ministry of National Guard - Health Affairs, Riyadh, SAU

**Keywords:** tetany with gastroenteritis, internal medicine (general medicine), acid-base disorder, alklosis, calcium, normocalcemia, tetany

## Abstract

Here, we report on a case of a 24-year-old female who is previously medically healthy. She was admitted with a case of gastroenteritis. During her hospitalization, we noticed that the patient's wrists and metacarpophalangeal (MCP) joints were flexed, with her bilateral proximal (PIP) and distal (DIP) interphalangeal joints extended. Furthermore, she exhibited positive Chvostek and Trousseau signs. Her clinical features were consistent with acute tetany; however, she was demonstrating normal levels of calcium. Other laboratory results were positive for severe metabolic alkalosis and hypophosphatemia. Her signs and symptoms resolved completely after one dose of intravenous calcium gluconate. This case is notable because tetany alongside normocalcemia is a rare condition. Therefore, the immediate management of such cases is crucial.

## Introduction

Tetany is characterized as a neuromuscular excitation, which can be interpreted by electromyography (EMG) using high-frequency repetitive waves. It usually begins with abnormal sensations, typically perioral paresthesia before involving the motor system, which causes the patient to develop stiffness, muscle spasms, and myalgia. This manifests in the forced adduction of the thumb with flexion of the metacarpophalangeal (MCP) joints and wrists as well as the extension of the fingers. Moreover, if left untreated, the symptoms can progress and cause acute autonomic manifestations, including bronchospasms, biliary colic, diaphoresis, seizures, or even death due to myocardial dysfunction [1].

Tetany is an uncommon condition, and most of the time, it is pathological due to current underlying etiologies. Hypocalcemia can cause tetany when a sudden drop occurs in the calcium level, specifically when the serum ionized calcium concentration falls below 4.3 mg/dL (1.1 mmol/L), which corresponds to a total serum calcium concentration of 7.0-7.5 mg/dL (1.8 to 1.9 mmol/L) [1]. However, additional causes of tetany include alkalosis, hypokalemia, and hypomagnesemia, while the presence of hypocalcemia and alkalosis can act synergistically to cause tetany [2].

In the absence of electrolyte imbalance, the onset of tetany is unusual and has only been reported in a few studies [3-5]. Thus, the treating physicians normally face serious dilemmas when dealing with such cases. In this paper, we report on a young female patient with normocalcemic tetany whose symptoms completely resolved after an intravenous infusion of calcium, despite the fact that her serum ionized calcium levels were already normal.

## Case presentation

This case study focuses on a 24-year-old female who was medically healthy and presented to the ED after experiencing three days of abdominal pain, vomiting more than 20 times per day, and expelling diarrhea six times per day. Her abdominal pain, which had begun three days previously, was experienced as a colicky pain that was diffused, mild to moderate, and non-radiating. Her diarrhea was watery and moderate to large in amount. There was no coffee ground vomiting, melena, or hematochezia. Her vomiting was of food contents, whereby she vomited whatever she ate over the past three days prior to her admission. Her symptoms started after she ate at a restaurant with her family; there were no similar symptoms in her family. On examination, her heart rate (HR) was 75 beats per minute, blood pressure (BP) was 108/55 mmHg, respiratory rate (RR) was 22 breaths per minute, oxygen saturation was 98% in room air, and afebrile with a temperature of 36.9 degrees Celsius. Her abdomen was soft and lax with mild epigastric tenderness on deep palpation with no palpable organomegaly or rebound tenderness. She was admitted to the acute medical unit under a diagnosis of gastroenteritis because of her inability to tolerate oral feeding; thus, she was started on IV fluids (normal saline 80 mL per hour) and metoclopramide. Her initial laboratory results are shown in Tables 1-2.

**Table 1:** CBC MCV: mean corpuscular volume, MCH: mean corpuscular hemoglobin, WBCs: white blood cells

Test	Result	Reference range
Hemoglobin (g/L)	122	120-160
MCV (fL)	82.4	76-96
MCH (pg)	26.5	27-32
WBCs (×10^9^/L)	3.39	4-11
Neutrophils count (×10^9^/L)	1.56	2-7.5
Platelets (×10^9^/L)	253	150-400

**Table 2:** Renal profile and electrolytes eGFR: estimated glomerular filtration rate, BUN: blood urea nitrogen

Test	Result	Reference range
eGFR (ml/min/1.73m^2^)	121	>60
Creatinine (μmol/L)	57	50-98
Serum bicarbonate (mEq/L)	17	22-29
BUN (mg/dL)	1.3	2.5-7.5
Adjusted calcium (mmol/L)	2.2	2.1-2.55
Sodium (mmol/L)	139	136-145
Potassium (mmol/L)	3.6	3.5-5.1
Phosphorus (mmol/L)	0.41	0.74-1.52
Magnesium (mmol/L)	0.70	0.66-1.07

**Figure 1:**
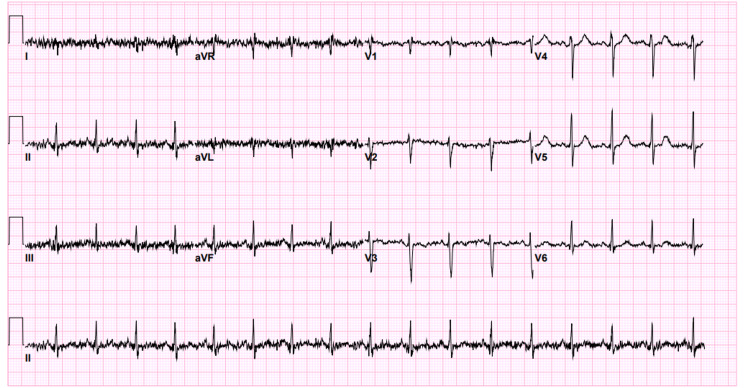
ECG Artifacts due to muscle twitching. Normal sinus rhythm with a ventricular rate of 102 beats per minute, PR interval of 114 ms, QRS duration of 74 ms, and QT/QTc of 340/443

Subsequently, the patient's symptoms improved the following day, and she was able to tolerate food administered orally without vomiting, and her diarrhea also improved. She was sent home with instructions to return to the ED if her symptoms worsened again. The patient was prescribed oral antiemetic and oral rehydration solution and subsequently discharged. Six hours after returning home, the patient again started to undergo persistent vomiting of whatever she consumed, and she returned to the ED. She was again admitted to the acute medical unit and was started on an IV normal saline at 80 mL per hour and an IV metoclopramide at 10 mg every eight hours. Four hours later, the primary nurse noticed that the patient had developed abnormal movements in her hand and face, and she immediately requested a re-assessment of the patient. During the re-assessment, the patient appeared anxious. She appeared alert and oriented to time, place, and people, although was complaining about an inability to keep her wrists and fingers extended in addition to numbness around her mouth and both eyes. On examination, the patient was tachypneic with a RR of 28 breaths per minute, tachycardic with an HR of 117 beats per minute, her BP was 113/58 mmHg, her oxygen saturation was 98% at room air, and her temperature was 37.1 degrees Celsius. Both of the patient's wrists and MCP joints were flexed with her bilateral PIP and DIP joints extended. Further examination showed that she exhibited positive Chvostek and Trousseau signs, bilateral periorbital paresthesia, circumoral numbness, and diffused muscle twitching in her lower limbs and back. No extrapyramidal signs were noted. A chest examination showed equal bilateral air entry with no added sounds and normal heart sounds with no audible murmurs. Her abdomen was soft and lax with mild tenderness from deep palpation. Owing to these findings, it was decided that the most probable explanation for the patient's presentation was acute tetany. Therefore, we immediately sent a request to the laboratory for her electrolyte and arterial blood gas (ABG) levels, and urine analysis, which is shown in Tables 3-4. ECG was also performed (Figure 1), and we arranged for brain-computed tomography.

**Table 3:** ABGs pH: potential of hydrogen, pCO_2_: partial pressure of carbon dioxide, pO_2_: partial pressure of oxygen, HCO_3_: bicarbonate, BE: base excess, P/F ratio: partial pressure of oxygen/fraction of inspired oxygen ratio, Na+: sodium, K+: potassium, Ca2+: calcium

Test	Result	Reference range
pH	7.605	7.35-7.45
pCO_2_ (mmHg)	11.3	35-45
pO_2_ (mmHg)	106.3	70-100
HCO_3_ (meq/L)	11.0	22-26
BE (mmol/L)	-6.2	-2-2
P/F ratio (mmHg)	506.4	>400
Na^+^ (mmol/L)	140.6	135-145
K^+^ (mmol/L)	3.2	3.5-5
Ca^2+^ (mmol/L)	1.283	1.15-1.30

**Table 4:** Urine analysis UA: urine analysis, RBC: red blood cell, WBC: white blood cell

Test	Result	Reference range
Specific gravity	1.017	1.015-1.025
UA ketones	80	Negative
UA blood (RBCs)	Negative	Negative
UA leukocyte esterase	Negative	Negative
UA WBC (WBC/hpf)	1	Negative
UA protein (mg/dL)	Negative	Negative
UA nitrite	Negative	Negative
UA RBC (RBC/hpf)	2	Negative

We decided not to wait for the laboratory results and to immediately administer 1 g of IV calcium gluconate, 2 g of IV magnesium sulfate, 30 milliequivalents (mEq) of IV potassium, and 20 millimoles (mmol) of IV sodium glycerophosphate. In anticipation of any further deterioration in the patient's condition, we involved the ICU team in the management of the patient. In an attempt to relieve the patient's anxiety, we asked her mother to sit beside her, and we explained to her that we would be around and that we would do our best to make her better, and anxiolytic was not given. Following the latest treatment option, the patient's condition gradually improved, and about 30 to 40 minutes later, the patient's symptoms and signs of tetany had totally resolved. The laboratory results that we had requested during the patient's acute tetany episode were shown later after intervention (Table 5).

**Table 5:** Laboratory results during the tetany episode WBCs: white blood cells

Test	Result	Reference range
Hemoglobin (g/L)	117	120-160
WBCs (×10^9^/L)	4.1	4-11
Platelets (×10^9^/L)	249	150-400
Adjusted calcium (mmol/L)	2.17	2.1-2.55
Sodium (mmol/L)	138	136-145
Potassium (mmol/L)	3.3	3.5-5.1
Phosphorus (mmol/L)	0.28	0.74-1.52
Magnesium (mmol/L)	0.67	0.66-1.07
Albumin (g/L)	40	34-54

The patient remained in the hospital for another three days and did not develop any further tetany episodes. She was started on 600 mg of calcium carbonate for three days, which was administered orally three times a day, and after her vomiting and diarrhea had shown signs of improvement, she was again discharged home in a stable condition, with close follow-up for possible EMG.

## Discussion

The early diagnosis and management of tetany is very important to prevent possible complications, which may include bronchospasm, laryngospasm, rhabdomyolysis, seizures, and arrhythmias. In our patient, a diagnosis was made depending on the clinical presentation, and the intervention was directed toward the most common causes of tetany, which include hypocalcemia, hypomagnesemia hypokalemia, and alkalosis [6]. Other conditions that may lead to tetany include recurrent vomiting, acute pancreatitis, anxiety with hyperventilation, vitamin D deficiency, hypoparathyroidism, and malabsorption disorders, such as celiac and Crohn’s diseases due to loss of gastrointestinal electrolytes (calcium, magnesium, potassium, and phosphorus), which are secondary to diarrhea [6-11]. The possible causes of tetany in our patient were multifactorial. She had anxiety and tachypnea, which led to respiratory alkalosis; she also had recurrent vomiting, which had occurred more than 20 times per day [8,9]. Metabolic and respiratory alkalosis increases the binding of ionized calcium to albumin and may cause tetany with normal total and adjusted calcium levels [9]. Furthermore, hypophosphatemia is a possible cause of tetany, as our patient presented with extremely low phosphorus levels [7]. Regardless of the laboratory samples that were sent and the subsequent results, our patient was exhibiting typical symptoms of tetany, including muscle cramps, paresthesia, carpopedal spasm, and circumoral numbness [10,11]. Thus, owing to these signs and symptoms, we began treatment options that targeted the most common causes of tetany, including hypocalcemia, hypomagnesemia, hypokalemia, and alkalosis. The patient demonstrated significant improvement and had a complete resolution of her symptoms after this intervention. The patient's ABGs (shown in Table 3) showed a mixed acid-base disorder consisting of respiratory alkalosis, normal anion gap metabolic acidosis (NAGMA), and high anion gap metabolic acidosis (HAGMA). The primary disorder was respiratory alkalosis, which manifested as a high pH and low CO2. The calculated anion gap was as high as 20 (high) and the calculated delta/delta ratio was 0.61, which means that there was a mixed disorder of normal and high anion gap metabolic acidosis. Respiratory alkalosis was due to the patient’s anxiety, NAGMA was most likely due to diarrhea, while HAGMA was most likely secondary to starvation ketoacidosis, which manifested as ketonuria (Table 4). Given that the patient's symptoms improved within 30 to 40 minutes after administering calcium gluconate (over 10 minutes) and after relieving the patient’s anxiety, the most likely explanation for the patient's symptoms and for this rapid improvement was either alkalosis, hypocalcemia, or both. She had improved prior to completing her potassium, magnesium, and phosphorus infusions (potassium infusion was over three hours, magnesium sulfate infusion was over two hours, and phosphorus infusion was over two hours). However, what made alkalosis the potential main cause of our patient’s diagnosis more than hypocalcemia was their normal ionized calcium level in ABG results (Table 3). Another result that indicated that hypocalcemia was unlikely compared to alkalosis was the patient's ECG result (Figure 1), which demonstrated a normal sinus rhythm, a normal ST segment, and a normal QTc interval (443 ms).

## Conclusions

Hypocalcemia is the most frequently described cause of tetany. In rare cases, tetany presented with normal calcium levels. Our patient presented with multifactorial normocalcemic tetany. Possible causes included anxiety, tachypnea, metabolic and respiratory alkalosis, and hypophosphatemia. An early diagnosis of tetany predominantly depends on recognizing the clinical signs and symptoms, while an early intervention targeting the most common causes leads to improved outcomes and the prevention of life-threatening consequences from tetany. In this patient, an intravenous infusion of calcium was a life-saving intervention that resulted in a total resolution of her symptoms.
